# Synchronization of networks of chaotic oscillators: Structural and dynamical datasets

**DOI:** 10.1016/j.dib.2016.03.097

**Published:** 2016-04-04

**Authors:** Ricardo Sevilla-Escoboza, Javier M. Buldú

**Affiliations:** a Centro Universitario de los Lagos, Universidad de Guadalajara, Jalisco 47460, Mexico; b Complex Systems Group & GISC, Universidad Rey Juan Carlos, 28933 Móstoles, Madrid, Spain; c Center for Biomedical Technology, UPM, 28223 Pozuelo de Alarcón, Madrid, Spain

**Keywords:** Nonlinear dynamics, Complex networks, Synchronization

## Abstract

We provide the topological structure of a series of *N*=28 Rössler chaotic oscillators diffusively coupled through one of its variables. The dynamics of the *y* variable describing the evolution of the individual nodes of the network are given for a wide range of coupling strengths. Datasets capture the transition from the unsynchronized behavior to the synchronized one, as a function of the coupling strength between oscillators. The fact that both the underlying topology of the system and the dynamics of the nodes are given together makes this dataset a suitable candidate to evaluate the interplay between functional and structural networks and serve as a benchmark to quantify the ability of a given algorithm to extract the structural network of connections from the observation of the dynamics of the nodes. At the same time, it is possible to use the dataset to analyze the different dynamical properties (randomness, complexity, reproducibility, etc.) of an ensemble of oscillators as a function of the coupling strength.

## Specifications table

**Table:** 

Subject area	Physics
More specific subject area	Nonlinear dynamics, complex networks, synchronization
Type of data	Tables, text files, graphs, figures
How data was acquired	We use a Multifunction Data Acquisition (DAQ), NI USB-6363 to acquire the signal of *N*=28 chaotic Rössler electronic circuits.
Data format	Raw
Experimental factors	Sampling rate: 37 KS/s; Number of bits: 16, relative time step 2.07E−5
Experimental features	Sampling of 28 Rössler-like chaotic oscillators coupled in a network configuration for different values o coupling strength
Data source location	Madrid, Spain
Data accessibility	Data is within this article

## Value of the data

• We provide the structural organization of a network of coupled oscillators and their corresponding dynamics. Thus, dataset can be used to quantify the ability of algorithms aiming to obtain the underlying structure of connections of a network from the dynamics of its nodes.
• Time series can be used as a benchmark to evaluate coordination/synchronization between chaotic oscillators as a function of the coupling and node topological properties.
• Randomness, complexity and other dynamical features of the time series can be extracted and compared with the topological properties of the nodes.
• Dataset can be used as experimental examples of synchronization (and the route to) of chaotic systems.

## 1 Data

All datasets are available at http://complexity.es/jmbuldu/data/dib_rse_jm*.* Specifically, we provide 5 experimental datasets, each one containing 101 items labeled TS_X.dat, where *X* takes values between 0 and 100, and corresponds to the minimum (*K*=0) and maximum coupling strength (*K*=1), respectively. The TS_X.dat files contain the *y* variable of the 28 nodes arranged in columns and with a length of 30,000 points. At the same time, the underlying structure of connections between chaotic oscillators is given in the file topology.dat.

**Fig. 1:**
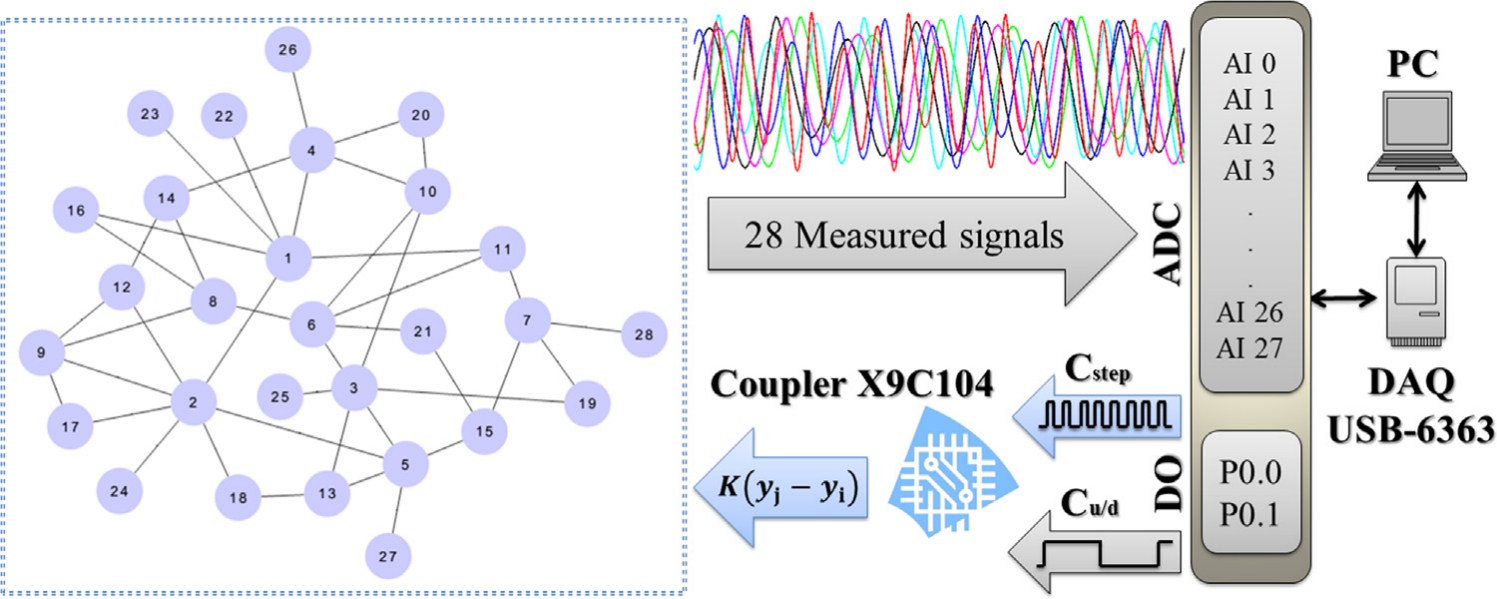
On the left, configuration of the actual network of physical connections between oscillators. On the right, qualitative description of the experimental setup.

**Fig. 2:**
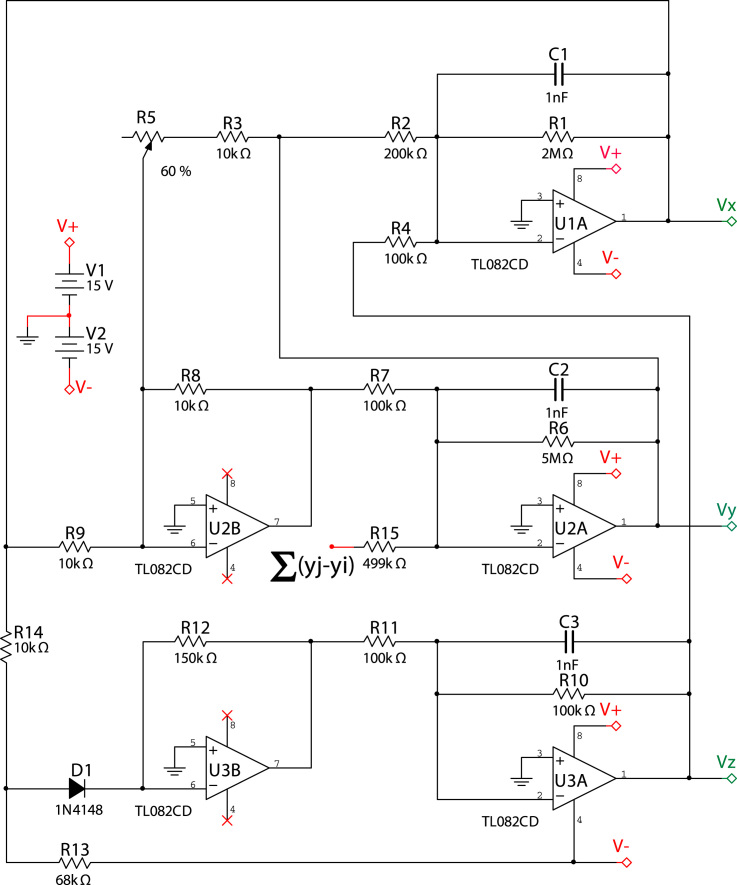
Electronic implementation of a Rössler-like electronic circuit [1], [2]. The values of the parameters of the electronic components are summarized in Table 1. The term $\sum \left(y_{j}-y_{i}\right)$ accounts for the diffusive coupling between units, whose corresponding electronic circuit is shown in Fig. 3.

The experimental setup reproduces the dynamics of a network of 28 Rössler-like oscillators [1], [2] (see Fig. 1 for details). The coupling is introduced through the *y* variable with coupling strength *K* so that the equations of motion become

(1) $${\overset{˙}{x}}_{i}=-\alpha_{1}\left(x_{i}+\beta y_{i}+\Gamma z_{i}\right)$$

(2) $${\dot{y}}_{i}=-\alpha_{2}\left({-\gamma x}_{i}+{\left[1-\delta \right]y}_{i}-K\phi \sum_{j=1}^{N}A_{\mathrm{ij}}\left[y_{j}-y_{i}\right]\right)$$

(3) $${\dot{z}}_{i}=-\alpha_{3}\left(-\eta G_{x_{i}}+z_{i}\right)$$

and the piecewise part is

(4) $$G_{x_{i}}=\left\{\begin{array}{ccc} 0 & \mathrm{if} & x_{i}\leq 3\\ \mu \left(x_{i}-3\right) & \mathrm{if} & x_{i}>3 \end{array}\right\}$$

where *α*_1_=500, *α*_2_=200, *α*_3_=1000, *β*=10, *Γ*=20, *γ*=50, *δ*=8.333, *μ*=15, *ϕ*=10, *K*=[0,1] and *A_ij_* is the structural connectivity matrix. It is possible to translate the Rössler-like equations into the electronic circuit shown in Fig. 2, which leads to the following equations of the system:

(5) $${\dot{x}}_{i}=-\frac{1}{R_{1}C_{1}}\left(x_{i}+\frac{R_{1}}{R_{2}}y_{i}+\frac{R_{1}}{R_{4}}z_{i}\right)$$

(6) $${\dot{y}}_{i}=-\frac{1}{R_{6}C_{2}}\left({-\frac{R_{6}R_{8}}{R_{9}R_{7}}x}_{i}+{\left[1-\frac{R_{6}R_{8}}{R_{C}R_{7}}\right]y}_{i}-K\frac{R_{6}}{R_{15}}\sum_{j=1}^{N}A_{\mathrm{ij}}\left[y_{j}-y_{i}\right]\right)$$

(7) $${\dot{z}}_{i}=-\frac{1}{R_{10}C_{3}}\left(-\frac{R_{10}}{R_{11}}G_{x_{i}}+z_{i}\right)$$

Here the piecewise part is

(8) $$G_{x_{i}}=\left\{\begin{array}{ccc} 0 & \mathrm{if} & x_{i}\leq I_{d}+I_{d}\frac{R_{14}}{R_{13}}+V_{\mathrm{ee}}\frac{R_{14}}{R_{13}}\\ \frac{R_{12}}{R_{14}}x_{i}-V_{\mathrm{ee}}\frac{R_{12}}{R_{13}}-I_{d}\left(\frac{R_{12}}{R_{13}}+\frac{R_{12}}{R_{14}}\right) & \mathrm{if} & x_{i}>I_{d}+I_{d}\frac{R_{14}}{R_{13}}+V_{\mathrm{ee}}\frac{R_{14}}{R_{13}} \end{array}\right\}$$

**Fig. 3:**
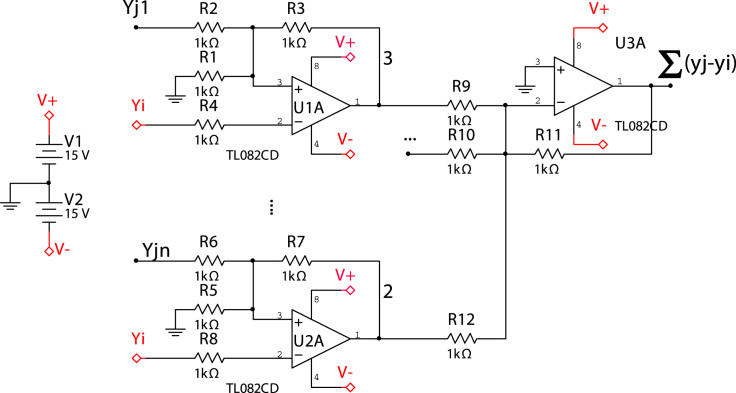
Electronic implementation of the diffusive coupling between a Rössler-like system and all of its neighbors. Each branch of the circuit accounts for the difference between oscillators *i* and *j,* being *j* each of its neighbors. Finally, a voltage adder joins the output of each branch (i.e., neighbors).

The values of the electronic components of the circuits are given at Table 1. Finally, Fig. 3 corresponds to the electronic circuit introducing the diffusive coupling between oscillators. Note that a digital potentiometer X9C104 allows adjusting the values of coupling strength (*K*).

## 2 Experimental design

The experimental setup is shown in Fig. 3 and it consists of an electronic array (EA), a multifunction data card (DAQ USB-6363), and a personal computer (PC). The EA comprises 28 Rössler-like oscillators, each node having an individual electronic coupler controlled by a digital potentiometer (9C104), which is adjusted through a digital output signal (DO) coming from ports P0.0 and P0.1. Port P0.0 is used to set the 28 values of the coupling resistance (adequately scaled to correspond to the values of *K*) and P0.1 increases or decreases the value of the resistance through a voltage divisor (the resolution allowing for 100 discretized steps). The full experimental process is controlled with a virtual interface developed in Labview 8.5 that can be considered as a state machine. The experimental procedure is realized as follows. First, *K* is set to zero, after a waiting a transient time of 500 ms (roughly corresponding to 600 cycles of the autonomous systems), the output signals of the 28 circuits are acquired by the analog ports (AI 0; AI 1; …; AI 27). Once the dynamics of the whole ensemble is recorded, the value of *K* is increased by one step (0.01), and the signals are again stored in the PC for further analysis. This process is repeated until the maximum value of *K* is reached.

## Figures and Tables

**Table 1:** Values of the electronic components used for the construction of the Rössler-like circuit given by Eqs. (5)–(7) and Eq. (8).

*C*_1_=1 nF	*C*_1_=1 nF	*C*_1_=1 nF	
*R*_1_=2 MΩ	*R*_2_=200 KΩ	*R*_3_=10 KΩ	*R*_4_=100 KΩ
*R*_5_=50 KΩ	*R*_6_=5 MΩ	*R*_7_=100 KΩ	*R*_8_=10 KΩ
*R*_9_=10 KΩ	*R*_10_=100 KΩ	*R*_11_=100 KΩ	*R*_12_=150 KΩ
*R*_1_=2 KΩ	*R*_14_=10 KΩ	*R*_15_=499 KΩ	*R_C_*=*R*_3_+*R*_5_
*I_d_*=0.7	*V_ee_*=15	K=[0−1]	
